# Case Report: First Case of Consolidation Immunotherapy After Definitive Chemoradiotherapy in Mediastinal Lymph Node Metastatic Sarcomatoid Carcinoma

**DOI:** 10.3389/fonc.2021.788856

**Published:** 2022-01-10

**Authors:** Yu Wang, Lin Yang, Jianyang Wang, Lin Gui, Wei Li, Zhiqiang Liu, Xiangyu Ma, Yin Yang, Luhua Wang, Nan Bi

**Affiliations:** ^1^ Department of Radiation Oncology, National Cancer Center/National Clinical Research Center for Cancer/Cancer Hospital, Chinese Academy of Medical Science and Peking Union Medical College, Beijing, China; ^2^ Department of Pathology, National Cancer Center/National Clinical Research Center for Cancer/Cancer Hospital, Chinese Academy of Medical Science and Peking Union Medical College, Beijing, China; ^3^ Department of Medical Oncology, National Cancer Center/National Clinical Research Center for Cancer/Cancer Hospital, Chinese Academy of Medical Sciences & Peking Union Medical College, Beijing Key Laboratory of Clinical Study on Anticancer Molecular Targeted Drugs, Beijing, China; ^4^ Department of Radiation Oncology, National Cancer Center/Cancer Hospital & Shenzhen Hospital, Chinese Academy of Medical Sciences and Peking Union Medical College, Shenzhen, China

**Keywords:** immunotherapy, chemoradiotherapy, PD-L1 inhibitors, case report, sarcomatoid carcinoma

## Abstract

Sarcomatoid carcinoma (SC) is a rare lung cancer subtype with poor prognosis and lack of effective treatment regimens. Studies concerning SC indicated common programmed death ligand-1 (PD-L1) overexpression and higher tumor mutational burden, leading to potential benefits from immunotherapy. The present case is the first report employing PD-L1 inhibitor durvalumab following definitive concurrent chemoradiotherapy (cCRT) in a patient with mediastinal lymph node metastatic SC, which was considered as a high probability of pulmonary origin but unclear primary lesion. After the 19-month follow-up, there was neither local recurrence nor distant metastasis. The patient was in a good condition, with the thoracic lesion controlled at Partial response-Response Evaluation Criteria in Solid Tumors (PR-RECIST). Except for grade 2 esophagitis, none of the other adverse events was observed. Our first attempt to adopt the consolidation immunotherapy after cCRT in unresectable locally advanced mediastinal SC exhibited improved local control, manageable safety, and potential survival benefits, representing a novel and promising therapeutic option for SC and encouraging further research exploration of this regimen in the future.

## Introduction

Pulmonary sarcomatoid carcinoma (SC) is a rare subtype of non-small-cell lung cancer (NSCLC) accounting for less than 1.0% of all lung cancers (1), which is characterized by early metastasis occurrence and insensitive to platinum-based chemotherapy (2), leading to poor prognosis and lack of effective treatment regimens. Although traditional targetable genetic driver mutations in Anaplastic Lymphoma Kinase (ALK) and Epidermal Growth Factor Receptor (EGRR) are observed as low levels in SC (3), the growing body of literature with respect to SC supports that higher programmed death ligand-1 (PD-L1) expression and tumor mutational burden (TMB) are common (4, 5), which represent higher response rates and potentially survival benefits under immune checkpoint inhibitor (ICI) treatment, especially PD-L1 inhibitors (6). For all these, the optimal treatment strategy for SC remains controversial.

The PACIFIC regimen, up to 12 months of consolidation durvalumab (PD-L1 inhibitor) treatment after curative-intent concurrent chemoradiotherapy (cCRT), exhibited sustained survival benefits in patients with locally advanced unresectable NSCLC, received global approvals, and thus became the standard of care (SoC) in this setting (7). Of note, although immunotherapy has improved cancer prognosis and changed the treatment landscape for several tumor types, no evidence regarding the efficacy or safety of applying the PACIFIC regimen specifically to SC is available heretofore.

Herein, we for the first time reported a patient with unresectable mediastinal lymph node metastatic SC, with probably pulmonary origin but unclear primary lesion, treated with SoC consolidation durvalumab after cCRT to describe this novel and potential therapeutic option for SC.

## Patient Information

In May 2018, a 53-year-old Asian male, with a 33-year smoking history, underwent the left lung bullae resection under thoracoscopy-assisted small incision operation at a local hospital. The operation was successful, and postoperative pathology confirmed benign pulmonary bullae lesion. In the subsequent follow-up, he was in a good condition and completed smoking cessation under the advice of his doctor. The patient had neither other previous medical history nor family history of cancer. However, 2 years later, he was admitted to our hospital on account of an abnormal space-occupying lesion in the mediastinum during a routine computed tomography (CT) follow-up without any symptoms.

## Clinical Findings

**Figure 1:**
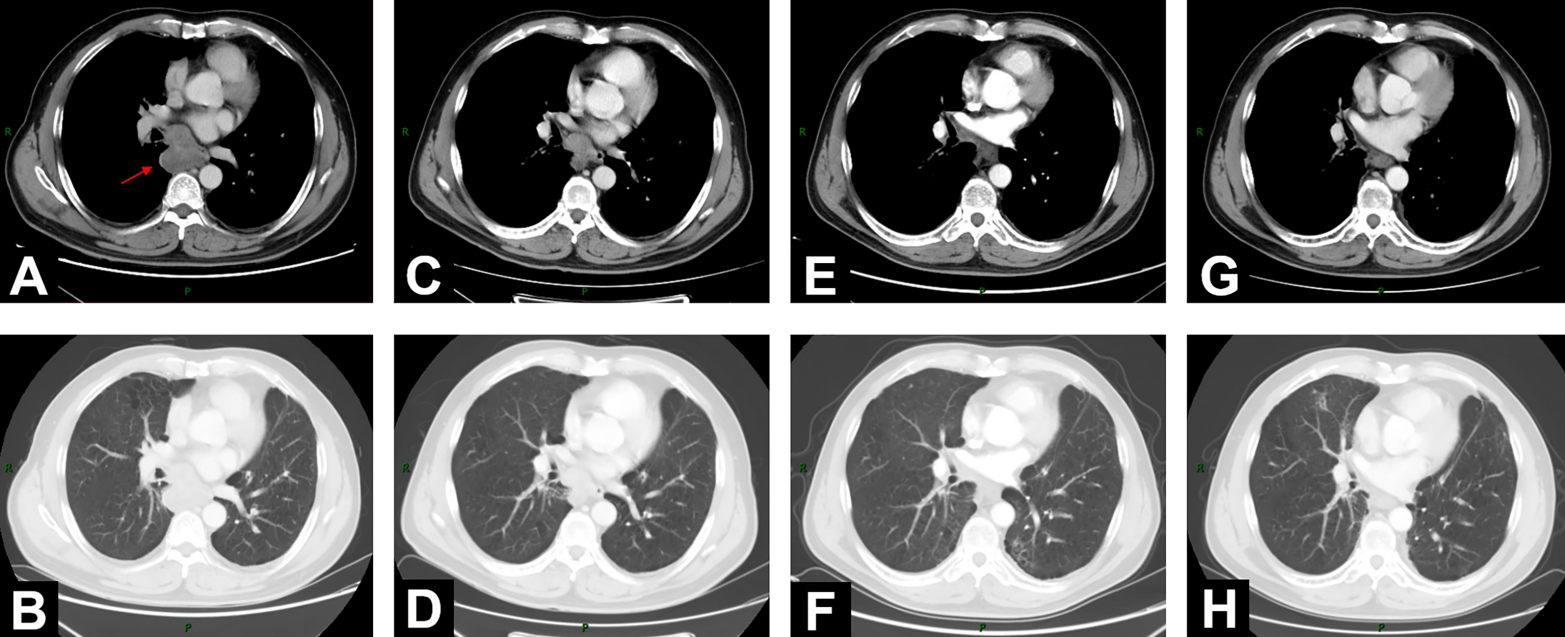
Changes of mediastinal lesion on chest contrast-enhanced CT scans. **(A, B)** On June 18, 2020, CT demonstrated enlarged mediastinal lymph node station 7 with heterogeneous enhancement (5.2 cm * 4.7 cm). **(C, D)** CT on August 11, 2020, after 2-cycle induction chemotherapy (3.9 cm * 2.9 cm). **(E, F)** CT on November 13, 2020 (2.0 cm * 1.5 cm), 1 month after concurrent chemoradiotherapy (cCRT). **(G, H)** At 1-year follow-up after consolidation durvalumab, CT on October 26, 2021 (1.6 cm * 1.3 cm).

**Figure 2:**
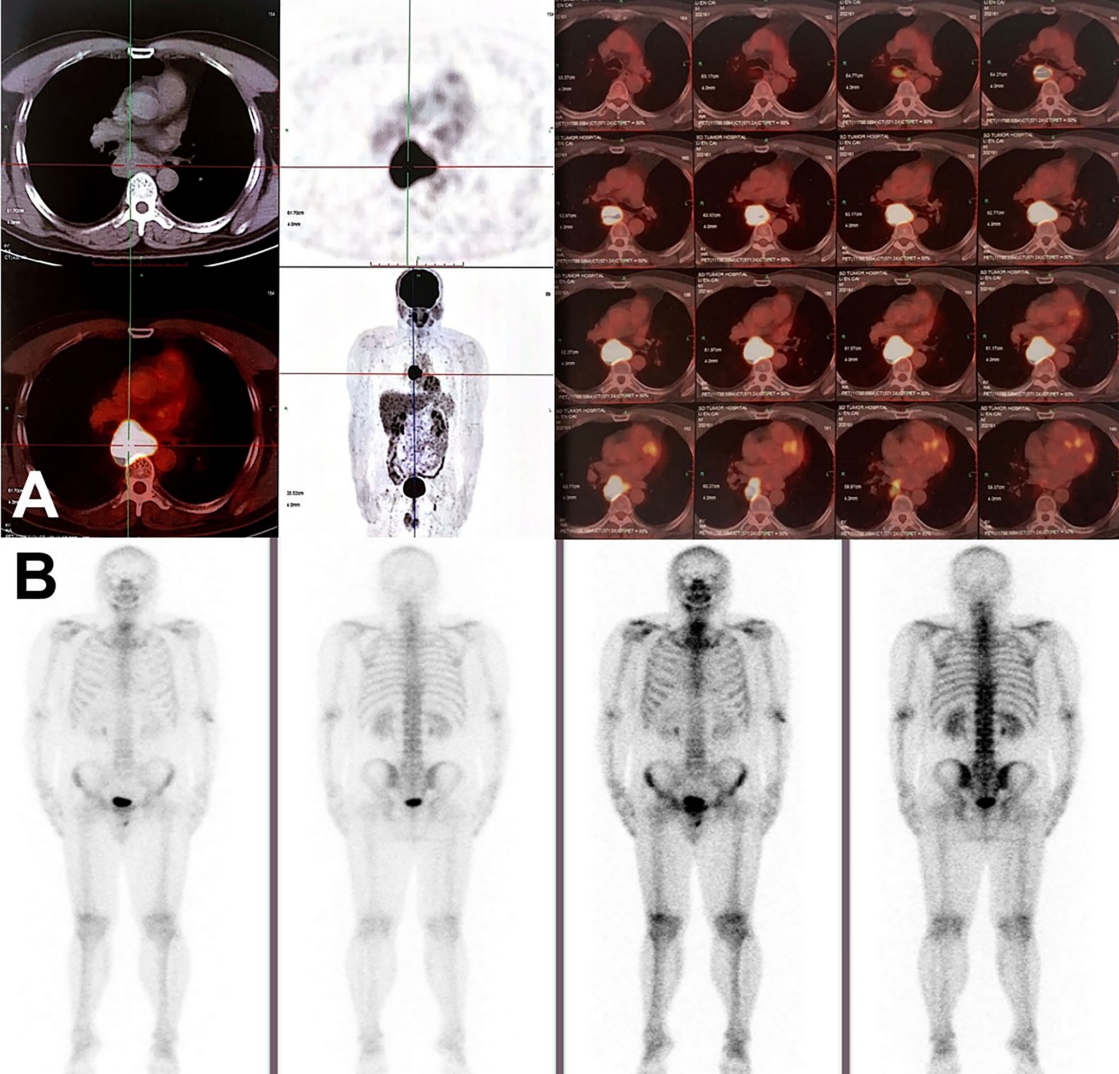
Radiographic findings on PET/CT and radioactive bone scan. **(A)** PET/CT scan in June 2020 showed the enlargement of mediastinal lymph node station 7 with max SUV 22.2, suggesting mediastinal lymph node metastasis. No visible primary pulmonary parenchymal focus but postoperative changes in the left lung. **(B)** No distant metastasis on radioactive bone scan.

On June 18, 2020, 2 years after the left lung bullae resection, CT scan demonstrated an irregularly enlarged mediastinal lymph node station 7 with heterogeneous enhancement, about 5.2 cm * 4.7 cm, considered as the metastasis, but no visible primary pulmonary parenchymal focus (**Figures 1A, B**). Subsequent bronchoscopy did not reveal any primary lung lesion either. Positron emission tomography (PET)/CT suggested the enlarged mediastinal lymph node station 7 with increased 18F-fluorodeoxyglucose (18F-FDG) uptake and unclear boundary with adjacent esophagus. The corresponding maximum standardized uptake value (SUV) of the enlargement was 22.2, and it was considered a metastasis possibly from a pulmonary origin, while no obvious pulmonary lesion with positive 18F-FDG uptake was found (**Figure 2A**). Hence, unexpectedly, the primary lesion remained unclear even after PET/CT. No evidence of distant metastasis emerged after performing PET/CT, brain MRI, and whole-body radioactive bone scan (**Figure 2B**). Physical examination (PE) also was negative.

## Timeline

**Figure 3:**
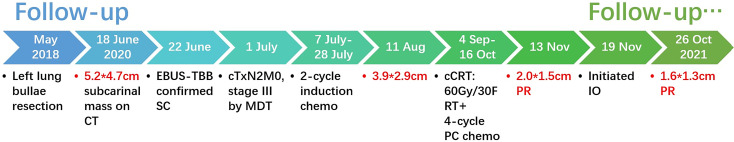
Timeline of the clinical events and treatment strategies. cm, centimeter; PR, partial response; Gy, gray; F, fraction; RT, radiotherapy; PC, nab-paclitaxel + carboplatin; IO, immunotherapy.

The timeline is presented in **Figure 3**.

## Diagnostic Assessment

**Figure 4:**
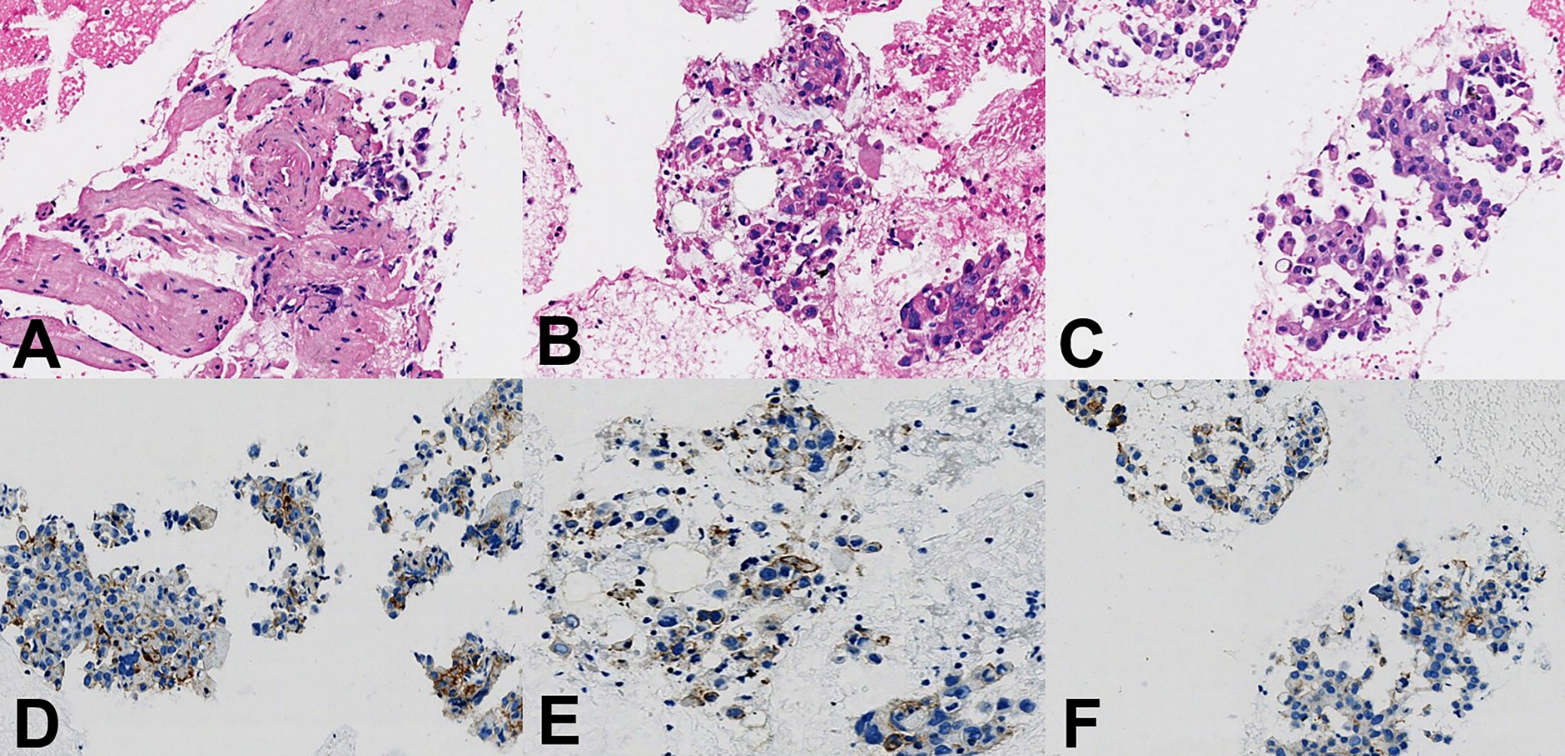
Histopathological findings. **(A–C)** Hematoxylin and eosin staining showed pleomorphic carcinoma with spindle and giant cells, ×400 magnification. **(D–F)** Immunohistochemistry staining to assess PD-L1 expression showed the tumor proportion score was 40%, ×400 magnification.

After a series of radiological examinations, the diagnosis was confirmed by the histopathological biopsies. Endobronchial ultrasonography (EBUS) inserted and advanced to the subcarinal mass and then EBUS-guided transbronchial biopsy (EBUS-TBB) confirmed *NSCLC, favor pleomorphic carcinoma* (**Figures 4A–C**). Immunohistochemistry (IHC) indicated *CK5/6 (+)*, *CK7 (+)*, *Syn (-)*, *CgA (-)*, *P40 (-)*, *CD5 (-)*, *CD117 (-)*, *NUT (-)*, *WT1 (-)*, *D2-40 (-)*, and *Ki67 (60%)*. The succeeding molecular testing showed negative results, including *EGFR/HER2/METex14 skipping/KRAS/NRAS/BRAF mutations (-)* and *ALK/ROS1/RET fusions (-)*. IHC staining to assess the expression of PD-L1 showed that the tumor proportion score (TPS) was 40% (**Figures 4D–F**).

This case was subsequently under a lengthy and full discussion by the multidisciplinary team (MDT), consisting of experienced clinicians from the departments of pathology, radiology, thoracic surgery, medical oncology, and radiation oncology. Given the aforementioned clinical evidence, including multiple radiological examinations, the pathologically confirmed pleomorphic carcinoma from NSCLC with high PD-L1 expression, and the left lung bullae resection history 2 years ago, through the MDT consultation, the final diagnosis was confirmed as mediastinal lymph node metastatic SC (pleomorphic carcinoma) with probably pulmonary origin but unclear primary lesion, stage III [cTxN2M0, American Joint Committee on Cancer (AJCC) 8th]. Thoracic surgeons also concluded that this mediastinal lesion was unresectable due to its ill-defined boundary with the adjacent esophagus and proximity to the pericardium. After the MDT discussions, it was unanimously agreed that the most appropriate treatment was definitive cCRT. The patient consented to adopt this regimen.

## Therapeutic Intervention

On August 11, 2020, after 2 cycles of induction chemotherapy (paclitaxel liposome 300 mg d1 + nedaplatin 140 mg d1/q21d), the patient was well tolerated with Eastern Cooperative Oncology Group performance status (ECOG-PS) score 0. Chest contrast-enhanced CT showed that the tumor size of the mediastinal lesion had reduced to 3.9 cm * 2.9 cm (**Figures 1C, D**). Subsequently, he received definitive cCRT from September 4 to October 16. Radiotherapy planning was made with a volumetric modulated arc therapy (VMAT) using *Pinnacle^3^* planning system. Here, 4D-CT positioning technology was used to reduce the setup error caused by respiratory movement (**Supplementary Figure S1A**). The gross tumor volume (GTV) enclosed the visibly enlarged mediastinal lymph node station 7. A 5-mm margin was given to GTV for creating the clinical tumor volume (CTV). Planning target volume (PTV) was generated using a uniform 5-mm expansion around CTV. The prescription dose was 6MV-X VMAT 95%PTV 60Gy/2Gy/30F (**Supplementary Figure S1**). Four cycles of PC regimen chemotherapy (nab-paclitaxel 90 mg + carboplatin 290 mg d1/qw) were concurrently given.

One month after cCRT, the mediastinal lesion had continuously decreased to 2.0 cm * 1.5 cm (**Figures 1E, F**), and the efficacy evaluated by CT was PR, according to RECIST 1.1 criteria. However, considering the still obscure boundary between this subcarinal lesion and esophagus after cCRT, the potential long-term toxicity of radiotherapy, and the high expression of PD-L1 (TPS was 40%), the appropriate further treatment for this patient was systemic consolidation immunotherapy rather than radical surgery. Therefore, on November 19, 2020, within 42 days after the end of cCRT, he initiated consolidation durvalumab (620 mg, q2w) for up to 12 months.

## Follow-Up and Outcomes

When the patient completed the 12-month PD-L1 inhibitor immunotherapy, he was in a stable condition with ECOG-PS score 0. On October 26, 2021, at a 1-year follow-up after durvalumab, chest CT scan showed a further reduction of the mediastinal lesion to 1.6 cm * 1.3 cm (**Figures 1G, H**). Neither local recurrence nor distant metastasis was observed. Apart from grade 2 esophagitis related, no other radiation-related or immune-related adverse events (irAEs) happened throughout the treatment and follow-up.

## Discussion

To the best of our knowledge, the present case is the first report employing consolidation immunotherapy after curative-intent cCRT in a patient with unresectable mediastinal lymph node metastatic SC with most probably pulmonary origin but unclear primary lesion, stage III (cTxN2M0, AJCC 8th). SC is a rare subtype of NSCLC that is resistant to conventional chemotherapy, and the prognosis is much poorer than that of other types of NSCLC (8). Traditional targetable genetic driver mutations were observed as low levels in SC (3), and the response to genetically targeted treatment, such as gefitinib, in SC patients even with EGFR mutation was still small and transient (9). Nevertheless, the growing body of recent literature regarding lung SC indicates common PD-L1 overexpression and higher TMB (4, 5), leading to potential clinical benefits under ICI treatment, albeit lacking solid evidence, such as a clear molecular mechanism pathway or phase 3 randomized controlled trials (RCTs). This case pertaining to the use of consolidation PD-L1 inhibitor durvalumab after cCRT (the PACIFIC regimen) in unresectable locally advanced SC exhibited improved patient outcomes and manageable safety. Meanwhile, PD-L1 expression is an important biomarker to predict the efficacy of immunotherapy. In this case, TPS was 40%, which not only matched the characteristics of PD-L1 overexpression in SC but also suggests the potential survival benefits from durvalumab for SC patients.

There are many case reports and retrospective analyses with respect to immunotherapy in SC, indicating positive therapeutic effects (6, 10–12), whereas the efficacy of combining cCRT with immunotherapy for SC patients was hardly discussed. Given the increasing attention paid to this combination regimen, our report applied this novel treatment to oncology practice for the first time and identified its potential value, encouraging further research exploration. The obvious benefits of combining radiotherapy with ICIs have been demonstrated by mechanism studies, as well as large-scale RCTs (13–15). Radiotherapy prior to immunotherapy would be likely to stimulate more robust and diverse immune cells, leading to more antigenic stimulation, presentation, and T-cell primers, thereby improving the effectiveness of ICI treatment (11). In addition, radiotherapy can strengthen local control, and thus the combination of radiotherapy and systematic immunotherapy tends to reduce both local recurrence and distant metastasis, further prolonging survival. In a word, ICIs following radiotherapy might be an improved and promising therapeutic option for patients with SC, especially since the significant survival benefits of this combination therapy in NSCLC patients has been proven by the PACIFIC trial (7, 15–18). Further investigations should be performed to explore the efficacy and safety of this combination regimen to optimize the treatment for SC.

The limitations of this report lie in the inconclusive diagnosis due to the unclear primary pulmonary lesion. However, the final diagnosis had been confirmed by the gold-standard histopathological examination and discussed through MDT consultation, which largely guaranteed the accuracy of the diagnosis. Besides, the 19-month follow-up is not enough, although the median survival time of lung SC was only 9.9 months (95% CI 7.6–12.6) from Mayo clinic experience (19). We will continue the long-term follow-up to document the prognosis of the PACIFIC regimen in this setting.

## Conclusions

Our first attempt to adopt consolidation immunology after definitive cCRT in locally advanced unresectable mediastinal lymph node metastatic SC exhibited improved local control, manageable safety, and potential survival benefits, representing a novel and promising therapeutic option for SC in the future and encouraging further investigations of the PACIFIC regimen in SC patients.

## Patient Perspective

The patient consented and adhered to the recommended treatment regimen. He and his family were not only satisfied with the improvement in his physical condition but also gratified by his recovered mental health.

## Data Availability Statement

The original contributions presented in the study are included in the article/**Supplementary Material**. Further inquiries can be directed to the corresponding author.

## Ethics Statement

Written informed consent was obtained from the individuals for the publication of any potentially identifiable images or data included in this article.

## Author Contributions

YW collected and analyzed clinical information and wrote the original draft. LY performed histopathological investigations and interpretation of results. JW, LG, and WL were the clinicians in charge of patient care and management. ZL and XM performed the radiotherapy planning and data analysis. YY contributed to conception and design of the study. LW and NB reviewed the article and contributed to the final draft. All authors contributed to the article and approved the submitted version.

## Funding

This work was supported by the National Key R&D Program of China (2018YFC1312104).

## Conflict of Interest

The authors declare that the research was conducted in the absence of any commercial or financial relationships that could be construed as a potential conflict of interest.

## Publisher’s Note

All claims expressed in this article are solely those of the authors and do not necessarily represent those of their affiliated organizations, or those of the publisher, the editors and the reviewers. Any product that may be evaluated in this article, or claim that may be made by its manufacturer, is not guaranteed or endorsed by the publisher.

## Supplementary Material

Supplementary Figure 1 Radiotherapy planning of volumetric modulated arc therapy (VMAT) using *Pinnacle^3^* planning system. **(A)**, Does distribution on 4D-CT. **(B)**, Contrast-enhanced CT. **(C)**, Dose-volume histogram of target volumes and organs at risk.
