# Supplementary figures and images for: Case Report: First Case of Consolidation Immunotherapy After Definitive Chemoradiotherapy in Mediastinal Lymph Node Metastatic Sarcomatoid Carcinoma
Source: Front Oncol. 2022 Jan 10;11:788856. doi: 10.3389/fonc.2021.788856 (PMC8785342; doi:10.3389/fonc.2021.788856)

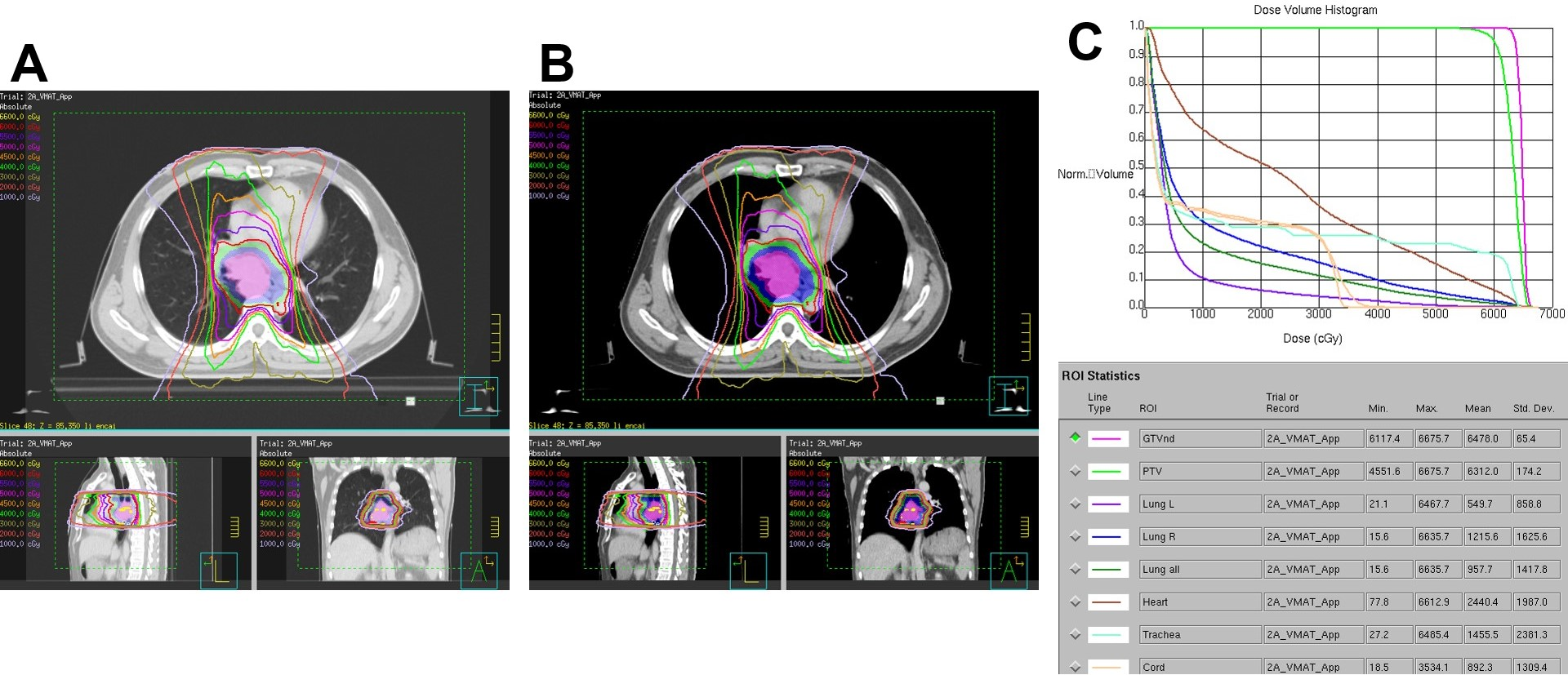

Supplement: Supplementary Figure 1 — Radiotherapy planning of volumetric modulated arc therapy (VMAT) using Pinnacle3 planning system. (A), Does distribution on 4D-CT. (B), Contrast-enhanced CT. (C), Dose-volume histogram of target volumes and organs at risk. [file Image_1.tiff]
